# Late Stage of Corneal Decompensation Caused by Progressive Keratoconus: Can We Treat It and Save the Cornea?

**DOI:** 10.1155/2015/795826

**Published:** 2015-04-30

**Authors:** Igor Knezović, Mirna Belovari Višnjić, Hrvoje Raguž

**Affiliations:** ^1^Department of Ophthalmology, Clinical Hospital Dubrava, 10000 Zagreb, Croatia; ^2^Department of Ophthalmology, General Hospital, 40000 Čakovec, Croatia; ^3^Knezović Vision Group Eye Center, 10000 Zagreb, Croatia

## Abstract

*Aim*. To report a case of 40-year-old male with progressive bilateral keratoconus who had undergone transepithelial phototherapeutic keratectomy (TE-PTK) and corneal collagen cross-linking (CXL) using hypoosmolar riboflavin solution in a same day procedure. *Methods*. Eye examination showed that UCDVA on both eyes was 0,01 according to Snellen charts, and slit lamp biomicroscopy showed paracentral diffuse intrastromal corneal haze. Anterior OCT marked stromal hyperreflective zones and localized paracentral thinning of the cornea. Scheimpflug tomography noted keratoconus stages III-IV on both eyes. After 40/35 microns TE-PTK, a CXL was performed for 30 minutes using hypoosmolar riboflavin solution. The left eye was treated first and the right eye 1 month after. Follow-up period was 10 months. *Results*. One month after the treatment both eyes showed improvement in corneal topography and the UCDVA was better. Eight months after the treatment BSCVA improved to 0,6 in both eyes using Rose K2 contact lenses and remained stable. *Conclusion*. TE-PTK and CXL using hypotonic riboflavin solution as a same day procedure have been shown to be a safe and promising method in this case of progressive keratoconus. It was necessary to consider certain parameters that could influence the safety and the final outcome of this combined protocol.

## 1. Introduction

Keratoconus is a corneal disease manifested by progressive thinning and ectasia which induces irregular astigmatism resulting in visual impairment and corneal blindness if not treated. Corneal collagen cross-linking (CXL) has become the standard for progressive keratoconus treatment after numerous clinical studies have proven its efficiency and safety in suitable eyes [1–5]. The standard protocol is suitable for eyes which have a minimum corneal thickness of 400 *μ*m, but some patients with keratoconus undergo more detailed examination with severely reduced visual acuity and corneal thickness less than 400 *μ*m. A few modifications of the standard procedure, such as transepithelial cross-linking [6], pachymetry-guided epithelial debridement before cross-linking [7], and the use of hypoosmolar riboflavin, have been suggested to benefit these patients without compromising their safety [8]. The patient in our case report had a severe corneal blindness due to keratoconus stages III-IV with extremely thinned cornea, which eliminated other corneal surgical procedures like intracorneal ring segment implantation [9] or partial topography guided photorefractive keratectomy (TG-PRK) [10]. His previous ophthalmologist presented him the penetrating keratoplasty [11] as the only possible treatment for this stage of keratoconus. The patient was highly motivated for the less invasive treatment, so we decided to perform a same day, combined transepithelial phototherapeutic keratectomy (TE-PTK) and corneal collagen cross-linking (CXL) using hypoosmolar riboflavin solution [5, 8] as a last attempt to delay a penetrating keratoplasty with its numerous complications [12].

## 2. Methods

**Figure 1:**
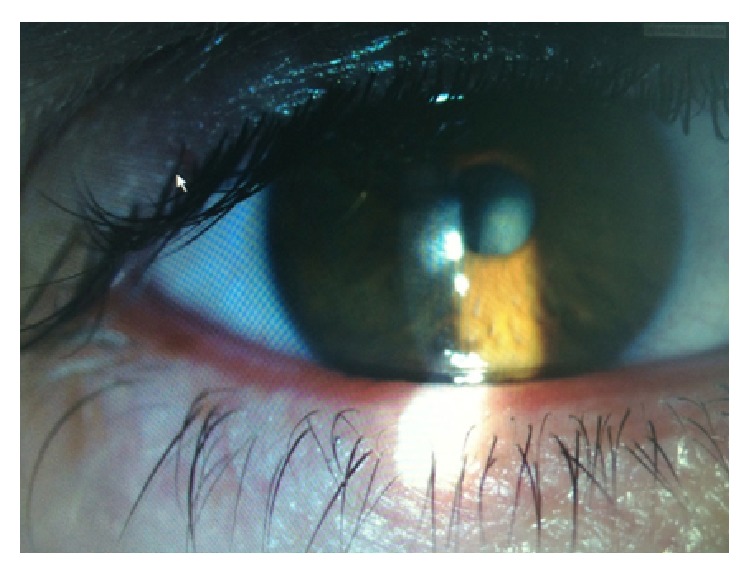
Slit lamp examination of the right eye.

**Figure 2:**
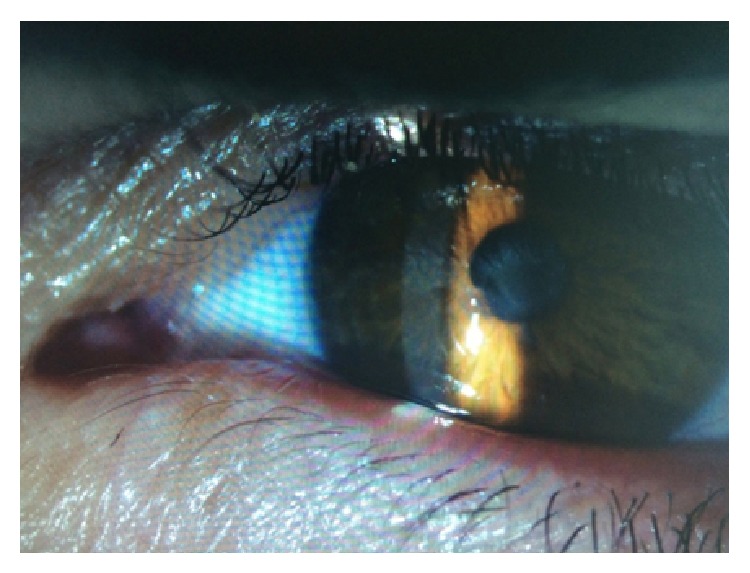
Slit lamp examination of the left eye.

**Figure 3:**
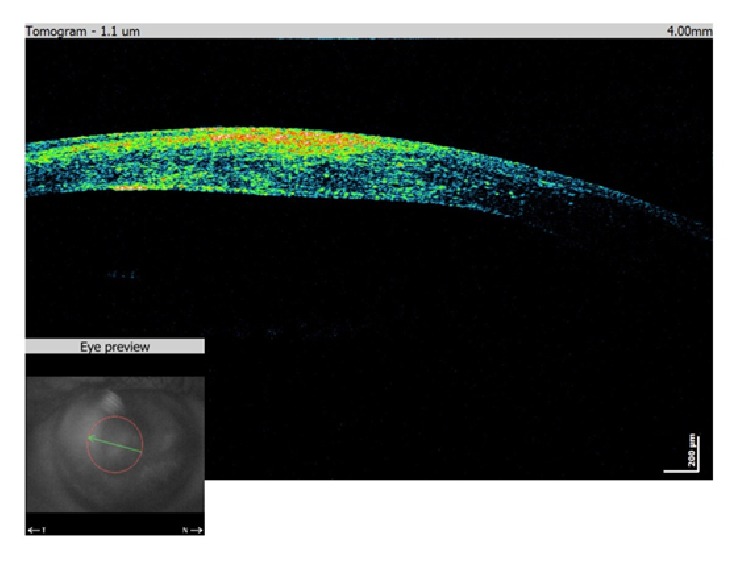
Preoperative SOCT anterior of the right eye.

**Figure 4:**
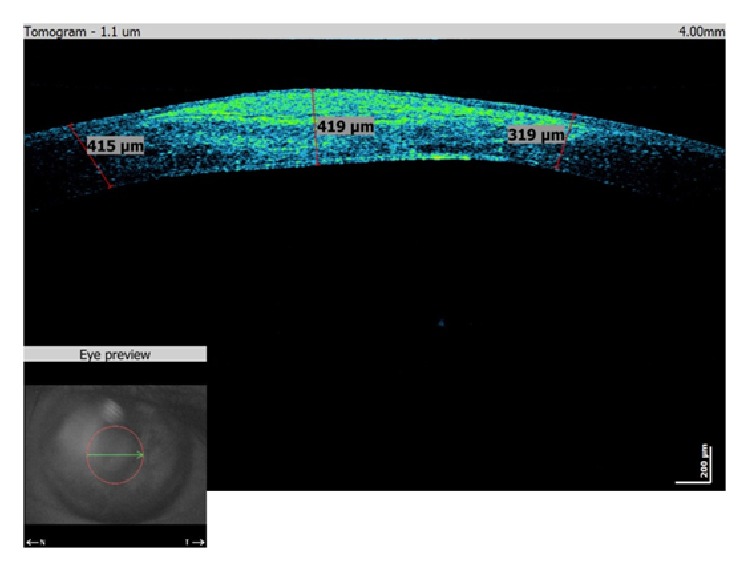
Preoperative SOCT anterior of the left eye.

**Figure 5:**
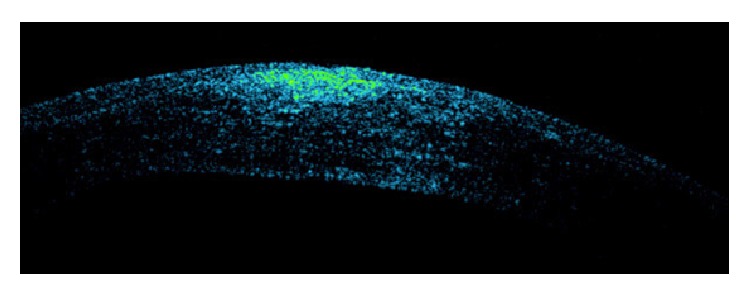
Anterior SOCT epithelium nonlinearity.

**Figure 6:**
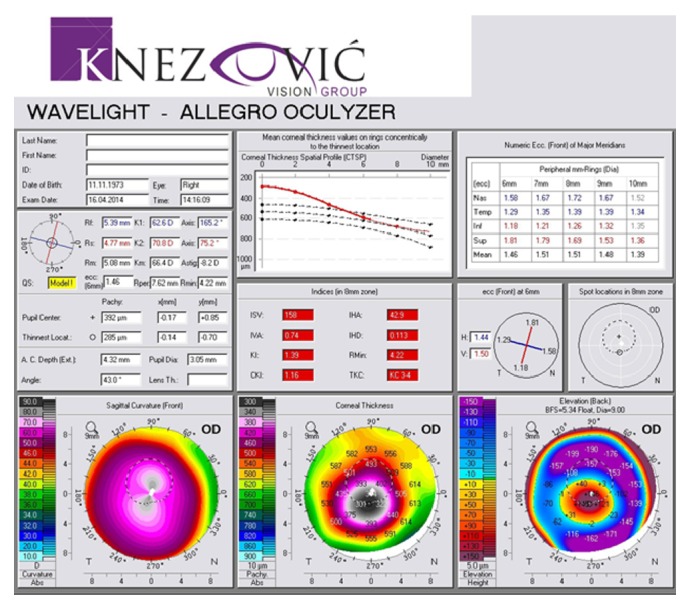
PCAM first examination, right eye.

**Figure 7:**
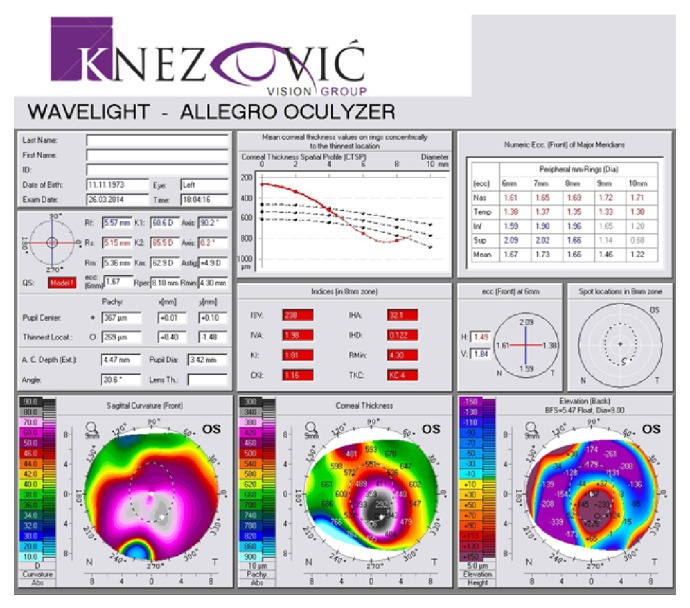
PCAM first examination, left eye.

**Figure 8:**
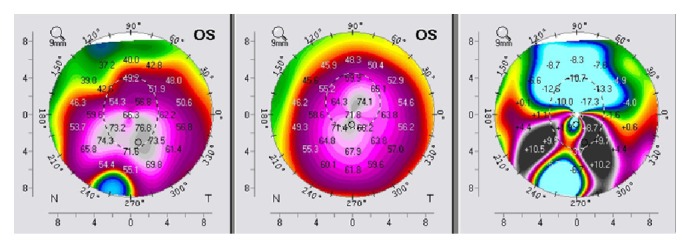
Scheimpflug tomography before and after the medicament therapy.

A 40-year-old man was sent to our eye center for further evaluation and treatment due to high degree of keratoconus and bilateral corneal blindness. His previous ocular history marked irregular astigmatism since adolescence, clinical signs of keratoconus appeared in early twenties, and visual acuity improvement was achieved by rigid gas permeable (RGP) contact lenses. A sudden decrease in visual acuity appeared three years ago in the left eye and one year ago in the right eye due to corneal decompensation and hydrops. At the time of examination, he was intolerant to RGP contact lenses due to pain and discomfort caused by wearing these lenses. We took a detailed eye examination with the following results: uncorrected distance visual acuity (UCDVA) on the right eye was 0,02 and on the left eye was 0,01; best spectacle corrected distance visual acuity (BSCDVA) in both eyes was between 0,05 and 0,1 according to Snellen charts. Preoperative slit-lamp biomicroscopy showed paracentral corneal scar after hydrops with diffuse intrastromal corneal haze (Figures 1 and 2). Endothelial cell count (specular microscope CEM 530, Nidek Co. Ltd., Japan) [13] was unmeasurable due to previous corneal decompensation and intrastromal corneal haze. Anterior spectral domain optical coherence tomography (SOCT Copernicus, Optopol Technology S.A., 42–400 Zawiercie, Poland) [14] marked in both eyes sharply bordered corneal intrastromal hyperreflective zones referring to previous corneal hydrops and stromal scars, bullous-shape subepithelial formation, and localized paracentral thinning of the cornea with highly irregular conic shape (Figures 3 and 4). Corneal epithelium thickness measurement showed a large difference in values from 25 to 55 microns (Figure 5). Scheimpflug tomography (Wavelight Allegro Oculyzer, Alcon Laboratories Inc.) noted keratoconus stages III-IV in the right eye and stage IV in the left eye according to Krumeich classification [15], and CCT 285/269 microns at the thinnest location. The front sagittal curvature map clearly shows a discrete asymmetric bow tie pattern in the right eye and irregular pattern in the left eye, and the corneal apex is decentrated inferotemporal. Back elevation map displayed a significant bilateral elevation more than +130 microns (Figures 6 and 7). During the month prior to surgery conservative therapy was conducted to prepare the corneal tissue for the treatment. Five percent hypertonic NaCl eye drops were used twenty times a day, dorzolamide-timolol eye drops twice a day, potassium iodine eye drops and preservative free artificial tears four times a day, and oral acetazolamide twice a day with a regular check and supplement of the potassium in the blood. This therapy resulted in decreased corneal curvature and reduction of the stromal hyperreflective zones and bullous-shape subepithelial formations which was confirmed by Scheimpflug tomography and SOCT anterior [16] (Figure 8).

## 3. Surgical Technique

The same procedure was performed on both eyes, first on the left eye and one month later on the right eye. The first step to prepare the patient for this combined procedure was to apply the topical anaesthetic oxybuprocaine hydrochloride 0,4% eye drops, and after that TE-PTK (Wavelight Allegretto Eye Q, 400 Hz device) was performed on central 7,0 mm zone with 40/35 (RE/LE) microns ablation depth for epithelium debridement. TE-PTK ablation depth was set to the mean between the thickest and thinnest corneal epithelium values, achieving thereby the primary correction of the corneal irregularity. Hypoosmolar riboflavin solution (0.1% riboflavin-5-phosphate is in the solution of 0.009% NaCl) was applied over the corneal surface every 2 minutes during the 30-minute period, to allow its absorption throughout the corneal stroma into the anterior chamber which was confirmed by slit lamp biomicroscopy. Ultrasound pachymetry was measured at the thinnest point of the cornea before and after 30 minutes of hypoosmolar riboflavin application and also intraoperative during the CXL every 5 minutes [17]. We noted a corneal swelling of 102 microns in the right eye and 107 microns in the left eye measured immediately prior to CXL. After the corneal alignment CXL was applied on corneal surface during the period of 30 minutes (UVA 370 nm light at an irradiance of 3,0 mW/cm²). Every 2 minutes during UVA exposure riboflavin was applied. Postoperatively, a bandage soft contact lens (Acuvue Oasys, senofilcon A) was placed on the eye for four days to provide the epithelium regeneration. Anti-inflammatory eye drops were administered after the procedure according to our protocol. Four months after the treatment soft toric contact lenses were used (Acuvue Oasys for astigmatism, senofilcon A) and eight months after the treatment specially designed Rose K2 lenses (Menicon Z, Dk 160) were fitted and used for visual acuity correction. The follow-up period was 10 months.

## 4. Results 

Outcome measurements included UCDVA and BCDVA according to Snellen charts, corneal topography registered by Scheimpflug camera, and anterior OCT evaluation of the corneal thickness, uniformity of the corneal epithelium, and demarcation line.

### 4.1. Visual Acuity and Refraction

One month after the treatment UCDVA was improved in both eyes between 0,1 and 0,15 according to Snellen charts. Four months after the treatment UCDVA enhanced from 0,15 to 0,2 in both eyes and BSCVA improved to 0,3 in right eye and 0,2 in left eye, using soft toric contact lenses. Eight months after the treatment specially designed, nontraumatic contact lenses for keratoconus (Rose K2, Menicon Z, Dk 160) were fitted improving BCDVA to 0,6 on both eyes.

### 4.2. Topographic Results

**Figure 9:**
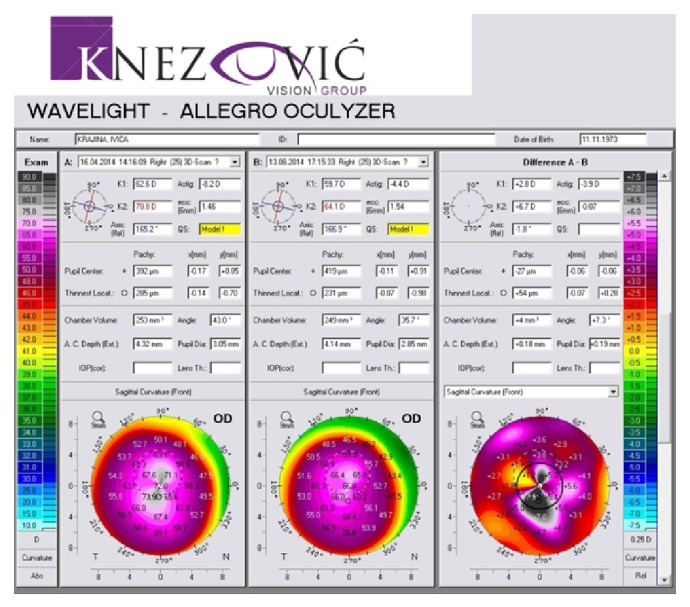
Pre- and postoperative Scheimpflug tomography (anterior sagittal curvature map), right eye.

**Figure 10:**
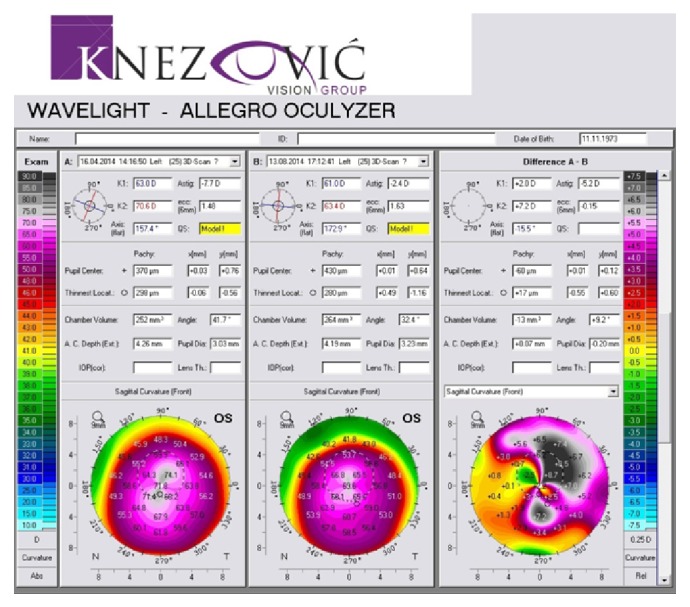
Pre- and postoperative Scheimpflug tomography (anterior sagittal curvature map), left eye.

**Figure 11:**
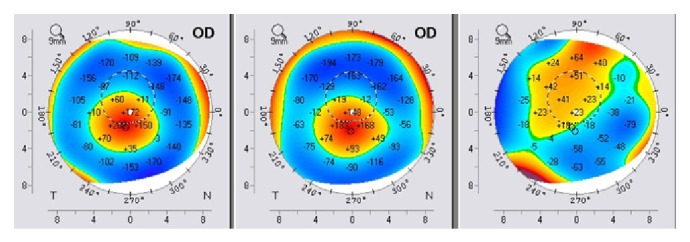
Pre- and postoperative Scheimpflug tomography (posterior elevation map), right eye.

**Figure 12:**
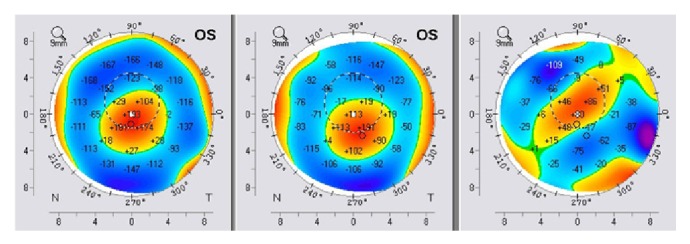
Pre- and postoperative Scheimpflug tomography (posterior elevation map), left eye.

Keratometric values were monitored before the procedure and after the follow-up period through the K1 and K2 values. We noticed significant changes in both K1 and K2 toward normal keratometric values compared before and after the follow-up period. K1/K2 values decreased from 62,6/70,8 D to 59,7/64,1 D in the right eye (Figure 9) and from 63,0/70,6 D to 61,0/63,4 D in the left eye (Figure 10). Each figure consists of three images. On the left side is the preoperative topography image and in the middle is the postoperative image, where remarkable topographic normalisation was noticed. On the right side is a difference map between preoperative and postoperative keratometric values. Topographic improvement was analysed in anterior sagittal curvature topographic and posterior elevation map using Wavelight Allegro Oculyzer. Figures 9 and 10 show remarkable topographic normalisation after the treatment with more regular pattern on both eyes, which is clearly visible on the anterior sagittal curvature comparative view. Figures 11 and 12 show posterior elevation comparative view which shows corneal stability after this combined protocol during the follow-up period.

### 4.3. SOCT Anterior Measurements

**Figure 13:**
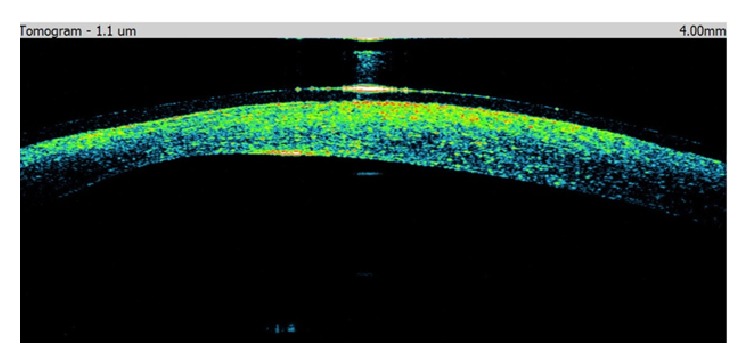
SOCT anterior: 1st postoperative day.

**Figure 14:**
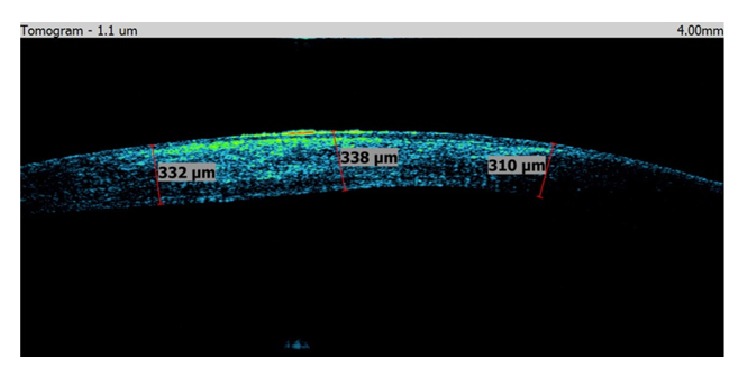
SOCT anterior: 10 months after treatment.

The first day after the treatment proves corneal tissue stability on the anterior SOCT view with a therapeutic soft contact lens placed over the cornea (Figure 13). During and after the follow-up period we noticed more regular corneal shape without large epithelium irregularities (Figure 14).

## 5. Discussion

During the UV light exposure, riboflavin protects the deeper ocular structures such as the corneal endothelium, lens, and retina from UV-A irradiances that are too high; it is also known that the combination of riboflavin and UV-A light contributes to 80–95% absorption into the cornea during cross-linking [18, 19]. To enhance CXL effect regarding keratometric and refractive outcomes, Kymionis et al. 2014 reported several refractive surgical techniques termed as “CXL plus.” As mentioned before, some of these combined procedures are not suitable for treating thin corneas because of extra tissue removal or limited stromal thickness (topography-guided photorefractive keratectomy and intrastromal corneal ring segments) while others (transepithelial phototherapeutic keratectomy and phakic intraocular lens) can be an option [20]. Wollensak et al. detected the damage threshold at the corneal endothelium to be 0.36 mW/cm^2^ (0.65 J/cm^2^); however, this intensity may cause damage if corneal thickness is below 400 *μ*m [1]. Numerous studies investigated intraoperative corneal thickness variations in keratoconic patients undergoing riboflavin/UV-A cross-linking treatment because this procedure has a lot of variations and it is not fully standardized with different preexposure imbibition time between 5 and 30 minutes [1, 21, 22]. A study by Baiocchi et al. 2009 on intrastromal concentration of riboflavin measured by high precision liquid chromatography (HPLC) pointed out that 10 minutes of preexposure imbibition is more than enough to ensure the optimal concentration of riboflavin in the stroma and protect the ocular structures before the cross-linking [23]. Also, Mazzotta and Caragiuli 2014 assessed intraoperative variations of corneal thinnest point in patients with Krumeich stage I or II progressive keratoconus and minimal corneal thickness greater than 400 microns using the standard riboflavin 0.1%-dextran 20% solutions registered by intraoperative optical pachymetry (Visante OCT, Zeiss, Jena, Germany). The most significant decrease in corneal thickness was noticed in the first 10 minutes of corneal soaking and the final value at the end of the operation was about 20% less than the initial value so they recommended that if a thickness under 380 microns is detected, the stroma should be reexpanded with hypotonic dextran-free riboflavin solution [24]. If cornea is already too thin before standard CXL, hypoosmolar riboflavin 0.1% can be used to induce the artificial swelling of the corneal tissue to at least 400 *μ*m: that way cytotoxic risk of UV-A to the endothelium can be reduced [25]. Kaya et al. 2012 evaluated corneal thickness during the CXL procedure by using combination of isoosmolar riboflavin solution with 20% dextran and hypoosmolar riboflavin solution without dextran in thin corneas (376.11 ± 19.88 after epithelial debridement) caused by corneal ectatic diseases. The thinnest pachymetric measurements decreased significantly after the application of isoosmolar riboflavin solution for 30 minutes and increased after hypoosmolar riboflavin application for 10 minutes. This study concluded that the iatrogenic swelling effect of the hypoosmolar riboflavin solution might be short acting and not steady throughout the surgery, so the better protective technique in thin corneas might be necessary [17]. Kymionis et al. 2012 pointed out the risks of using the standard CXL protocol in corneas less than 400 *μ*m after epithelial debridement and noticed a significant decrease in endothelial cell count in 14 eyes when using the standard isoosmolar CXL protocol [26]. Mazzotta and Ramovecchi 2014 presented a new surgical option for treating thin corneas known as epithelial island cross-linking technique. Partial epithelial islands on corneal thinnest point area left together with intrastromal riboflavin shield make a safe barrier for corneal endothelium protection [27]. Hafezi 2011 reported a failure of collagen cross-linking with hypoosmolar riboflavin solution in an extremely thin cornea and concluded that the increase in biomechanical resistance was not sufficient to stop the progression of the disease; therefore, minimal preoperative stromal thickness of 330 *μ*m needs to be a limit for a successful CXL procedure [28]. According to these observations, we performed ultrasound pachymetry at the thinnest point of the cornea before and after 30 minutes of hypoosmolar riboflavin application and also intraoperative during the CXL every 5 minutes [17]. Other studies proposed new iso-oncotic solutions as a possible option for the thin corneas treatment [29]. Raiskup and Spoerl reported stabilization of ectasia in corneas thinner than 400 microns after one-year study conducted on 32 eyes. They applied hypoosmolar riboflavin 0.1% solution every 2 min for 30 minutes, and during the CXL also hypoosmolar riboflavin drops were applied every 2 minutes [30]. Guided by their experience we recommended our patient a combined procedure, TE-PTK and hypoosmolar riboflavin CXL, hoping to improve the situation. This particular patient was thoroughly informed about the potential risks and complications, and he was highly motivated for the treatment. Phototherapeutic ablation was performed in attempt to normalize the highly irregular corneal surface (Cretan protocol) [5] and corneal collagen cross-linking to stabilize and improve biomechanical property in already thinned cornea. We noted improvement in all evaluated parameters during the 10-month follow-up period and the patient was able to continue with daily activities.

## 6. Conclusion

This case presents a success of combined TE PTK and CXL procedures using hypoosmolar riboflavin solution to achieve stabilization of high degree corneal decompensation caused by progressive keratoconus. Being aware of the fact that there is no ideal indication to this kind of treatment as in early stages of keratoconus with minimum thickness of at least 400 microns, we believe that this procedure could be an attempt to certain number of similar cases to prevent or postpone other surgical options like lamellar of penetrating keratoplasty. Further studies with larger number of patients and longer follow-up periods are mandatory to establish safety and effectiveness of this procedure.
